# Prevalence and risk factors for nonconsensual distribution of intimate images among Italian young adults: Implications for prevention and intervention

**DOI:** 10.1016/j.ijchp.2023.100414

**Published:** 2023-09-21

**Authors:** Antonella Brighi, Alberto Amadori, Kolis Summerer, Damiano Menin

**Affiliations:** a Free University of Bozen, Italy; b University of Ferrara, Italy

**Keywords:** Nonconsensual image distribution, Intimate partner violence, Impulsivity, Sexting, Decision-making

## Abstract

Nonconsensual distribution of intimate images (NCII), also known as revenge porn, has become a significant social issue in recent years, with severe consequences for victims. The present study aimed to investigate the prevalence and predictors of NCII victimization among young Italian adults, focusing on the role of sexting, intimate partner violence (IPV), impulsivity, and self-disclosure. An online survey was conducted among a sample of 2047 Italian young adults (*F* = 29.3 %, *M* = 53.4 %, Not Indicated=16.9 %; mean age = 24.4, SD = 4.4) using a convenience sample recruited through internet. The survey included questions on NCII victimization, sexting behavior, sextortion, and IPV. Our findings showed that 33.9 % of respondents reported engaging in sexting behavior, with females being three times more likely to engage in sexting than males. Furthermore, 3.3 % of participants reported being victims of NCII, with over one-third of victims experiencing three or more types of NCII victimization. Multiple regression analysis revealed that sexting and IPV were significant predictors of NCII victimization, and the interaction effect between self-disclosure and impulsivity was also a significant predictor. This study contributes to the understanding of NCII in Italy and highlights the need for interventions and prevention strategies to address both NCII and IPV, given their strong continuity. The results also suggest that the relationship between self-disclosure, impulsivity, IPV, and NCII victimization is complex and requires further investigation, suggesting a scenario where the climate of violence may impair the victim's decision-making.

## Introduction

Non-Consensual distribution of Intimate Images (NCII), also known as "revenge porn", is a growing and concerning phenomenon that affects individuals worldwide. Despite increasing awareness and legislative efforts aimed at combating this issue, victims continue to face significant emotional, social, and professional consequences as a result of their victimization. In 2021, a high-profile case of NCII was reported in Italy, involving a teacher fired from her job after intimate images of her were circulated online without her consent.

To date, limited research has examined the risk factors associated with NCII victimization. Given the potential consequences of NCII for individuals and society, it is critical to gain a better understanding of the factors that contribute to this phenomenon. The present study aims to address this gap in the literature by investigating the role of sexting and Intimate Partner Violence (IPV) as predictors of NCII victimization among a sample of Italian adults. By identifying the primary factors that increase the risk of NCII victimization, this study can contribute to developing effective interventions and prevention strategies to reduce the incidence of this harmful behavior.

### NCII definition

NCII refers to sharing sexually explicit or intimate photos or videos of individuals without consent (McGlynn et al., 2017; Said & McNealey, 2022). The use of the term "revenge porn" to describe NCII does not always accurately capture its diverse motivations, which can range from seeking social validation and sexual pleasure to practical jokes (Henry et al., 2019). Moreover, "revenge porn" implies that the victim did something to warrant the perpetrator's retaliation, which can contribute to victim blaming (Campbell et al., 2020). Thus, it is more fitting to emphasize the coercive nature of NCII rather than any implied notion of revenge.

The history of NCII can be traced back to the early days of the Internet when message boards and forums made it possible for individuals to share images anonymously. However, it was only with the advent of social media and the widespread use of smartphones that the problem became more prevalent (Ehman & Gross, 2019).

The characteristics of NCII are varied but typically involve sharing sexually explicit or intimate photos or videos without the consent of the person depicted. The images are often shared on social media platforms, message boards, through text messages or email (Henry & Powell, 2018; Karasavva et al., 2021; Walker et al., 2021). NCII commonly involves sextortion, where individuals are coerced, manipulated, or threatened to share explicit images or videos. Beyond sextortion, NCII experiences encompass non-consensual pornography, cyberbullying (including unauthorized webcam activation), and the illicit disclosure of private content through diverse methods. In all these instances, NCII underscores the absence or manipulation of consent in content distribution.

The repercussions of NCII can be severe, with victims often experiencing emotional distress, loss of privacy, and damage to their reputations (Henry et al., 2020; Powell et al., 2019). It can sometimes lead to bullying, harassment, and even physical harm (Bates, 2017a; McGlynn et al., 2017).

### Legal perspective in EU and Italy

In Europe, the legal response to the NCII varies by country. However, many states have laws that criminalize the distribution of sexually explicit or intimate images without consent (Mania, 2022). For example, in the United Kingdom, the Criminal Justice and Courts Act 2015 makes it illegal to disclose private sexual photographs or films intending to cause distress (Yar & Drew, 2019). Italy has outlined legal responses to NCII in its illegal and criminal procedure codes since 2019, criminalizing the distribution of images depicting sexual acts or intimate body parts without consent through Article 612-ter, which entails penalties including fines and imprisonment for up to three years. The punishment for this crime is a fine and a prison term of up to three years. Also, the Italian Civil Code protects personal image and reputation, a fundamental right protected by the Italian Constitution (Pavan & Lavorgna, 2021). It is important to note that the legal framework concerning NCII in the EU continually evolves, with some countries yet to implement specific laws addressing this issue (Caletti, 2021).

Interpretation and enforcement of these laws can also vary considerably across different jurisdictions. Moreover, the EU's General Data Protection Regulation (GDPR) comes into play when personal data, such as intimate images, is involved, offering protection and specific rights to individuals, including the right to have their data erased.

### Intimate partner violence and its association with NCII

Recent studies have unveiled a robust positive correlation between IPV and NCII (Eaton et al., 2020; McGlynn et al., 2017; Ruvalcaba et al., 2020). In many cases, NCII serves as a tool for control and manipulation within intimate relationships (Henry et al., 2019). IPV encompasses physical, sexual, psychological, or economic harm within intimate relationships (Finkel & Eckhardt, 2013; Renzetti & Edleson, 2008), manifesting as physical abuse, sexual abuse, emotional abuse, and controlling behavior (Hamby, 2014; Outlaw, 2009; World Health Organization, 2012). Abusers may employ the threat of sharing intimate images to manipulate their partner's behavior or hinder them from leaving the relationship. Sometimes, they follow through on the threat, sharing images as an act of vengeance or punishment. Moreover, IPV victims may face an increased risk of having their intimate images shared without consent (Eaton et al., 2020). Abusive partners may take and share intimate images without the victim's knowledge or consent, or use images taken consensually during the relationship to control or harm the victim. The emotional distress resulting from violating trust and privacy, coupled with the threat of additional abuse and harassment, underscores the severe and enduring impact of NCII within the context of IPV (Henry & Powell, 2015, 2018).

While understanding the associations between IPV and NCII is vital, exploring how individuals cope with the aftermath of NCII victimization is equally crucial. Coping strategies play a critical role in shaping the experiences and recovery of victims (Compas et al., 1993; Lazarus, 2006). However, a dearth of research has been dedicated to coping strategies in the context of NCII (Bates, 2017b; Wright, 2016). This gap in the literature has prompted our study to investigate the various coping strategies utilized by individuals who have experienced NCII victimization.

Coping strategies can encompass a wide range of responses, including seeking support from friends or family, confiding in someone they trust, discussing their experiences with others who have had similar encounters, or even taking legal action. The effectiveness of these coping strategies and their impact on the psychological well-being of NCII victims remain areas of inquiry.

### Sexting and online self-disclosure

Consensual sexting refers to the practice of sending sexually suggestive, nude, or nearly nude photos, videos, or messages through electronic means, with the mutual understanding and agreement of all parties involved (Barroso et al., 2022; Drouin et al., 2014; Mori et al., 2020). In these scenarios, individuals share intimate images with romantic partners or friends, expecting privacy and trust. On the other hand, non-consensual sexting pertains to sharing sexually explicit or suggestive messages or images without the depicted person's consent. Research indicates the prevalence of sexting among young adults and is often associated with risky behaviors like substance use, unprotected sex, and negative mental health outcomes (Clancy et al., 2019; Kosenko et al., 2017; Wachs et al., 2021). Although rates of sexting vary between studies, one meta-analysis reported that among young adults, approximately 14.8 % send sexts and 27.4 % receive them (Madigan et al., 2018), while another study indicates that sexual minority youth exhibit a higher propensity to participate in sexting activities (Mori et al., 2022).

A large corpus of literature indicates a strong and complex relationship between NCII and sexting, as the last one might occur both consensually and non-consensually (Englander & McCoy, 2017; Gámez-Guadix et al., 2022; Mori et al., 2020; Naezer & van Oosterhout, 2020; Said & McNealey, 2022). This implies that individuals engaging in sexting may face an elevated risk of NCII victimization, which can have profound emotional, psychological, and social consequences.

Sexting can also be a facet of cyberbullying and cyberstalking, contributing to NCII (Thulin et al., 2023). Nonetheless, it's important to recognize that sexting can allow adolescents to explore and express their sexuality consensually and safely (Kerstens & Stol, 2014; Naezer & Ringrose, 2018). Concurrently, research has explored the role of self-disclosure in sexting behavior, which involves voluntarily sharing personal information or images with others, expecting trust and privacy (Walsh et al., 2020). Recent findings highlight online self-disclosure as a complex decision-making process influenced by rational and emotional factors (Ostendorf et al., 2022). This suggests that individuals may not always make rational decisions regarding online self-disclosure, with dynamic factors like impulsivity possibly playing a role. However, despite these separate lines of investigation into sexting and self-disclosure, a critical gap remains in understanding how these factors intersect with NCII victimization.

### NCII and impulsivity

Impulsivity is a personality trait characterized by a tendency to act on impulse, often without careful consideration of consequences. This trait has been associated with various forms of risky behaviors, including NCII, both as perpetrators and victims (Chester & DeWall, 2018; Pina et al., 2017; Poythress & Hall, 2011).

Research suggests that individuals with high impulsivity scores are more likely to engage in NCII. For instance, a study by Döring (2014) found a positive association between impulsivity and the likelihood of sharing explicit images of a past partner without regard for potential consequences. Impulsivity can also be linked to NCII through sexting (Turban et al., 2020).

Moreover, impulsive individuals may be more prone to other online and offline risky behaviors (Partin et al., 2022), such as substance use, which can impair judgment and increase the likelihood of becoming victims of NCII.

Individuals with high impulsivity tend to use mobile internet options frequently for personal and professional purposes, displaying attentional patterns that indicate a propensity for less cautious decisions regarding security (Jeske et al., 2016). Notably, impulsivity can also be a symptom of other underlying mental health conditions like ADHD or BPD, potentially increasing the likelihood of engaging in NCII (Bőthe et al., 2019; Turban et al., 2020). Impulsivity is also associated with lower self-regulation and sensation seeking, resulting in problematic Internet usage (Billieux, 2012). Furthermore, impulsivity is not a static trait but rather dynamic, influenced by factors like stress and the environment. It can hinder decision-making by leading individuals to make quick, impulsive decisions without thorough consideration of options or potential risks and benefits (Franken et al., 2008; Martin & Potts, 2009; Wittmann & Paulus, 2008).

Being a victim of violence and NCII might significantly impact a person's decision-making abilities. The trauma and emotional distress caused by these experiences can affect a person's cognitive and emotional processing, making it harder for them to think clearly, regulate their emotions, and make sound decisions (Ceschi et al., 2014; Kim & Choi, 2020; Tull et al., 2007). Within the context of victimization, impulsivity may serve as a coping mechanism or response to feelings of powerlessness, loss of control, or emotional dysregulation (Kim & Choi, 2020; Marshall-Berenz et al., 2011; Weiss et al., 2020;Weiss et al., 2023).

Victims might make impulsive decisions to regain control, cope with overwhelming emotions, or seek revenge (Averdijk et al., 2016).

Moreover, impulsivity, as a decision-making style, may not yield direct consequences but can exacerbate certain behaviors, such as online self-disclosure, making them more problematic than they would otherwise be.

However, it is essential to acknowledge that impulsivity rarely acts in isolation and often interacts with other risk factors. Studies have demonstrated that impulsivity can act as a catalyst, amplifying the effects of both individual characteristics (Fox et al., 2010; Hamilton et al., 2014; Lightsey & Hulsey, 2002) and environmental factors (Auger et al., 2010; Lynam et al., 2000). Therefore, investigating the interaction effect of impulsivity with other risky behaviors is essential for comprehending its unique impact on human behavior.

While these factors may be explored in relation to NCII victimization, it is crucial to emphasize that the intent of this study is not to blame victims for their experiences. Instead, we aim to investigate the complex interplay of various factors related to victims’ online behaviors and decision-making within the context of NCII victimization. We recognize that perpetrators are solely responsible for their actions, and our study seeks to contribute to a deeper understanding of the multifaceted dynamics surrounding NCII.

Furthermore, it is important to clarify that our study adopts a victim-centered approach, placing utmost importance on the responsibility of perpetrators for their actions. The Routine/Lifestyle Activities Theory (RLAT; Cohen & Felson, 1979; Felson & Santos, 2010) provides a valuable framework for understanding how situational factors can heighten an individual's vulnerability to NCII victimization without placing blame on the victims themselves. It suggests that crime occurs when a motivated offender encounters a suitable target without capable guardianship within the context of their routine activities. In line with contemporary victimology perspectives (Clay-Warner & Edgemon, 2020; Fitz-Gibbon & Walklate, 2018; Jaishankar, 2020), we assert that victim-blaming is both ethically unsound and counterproductive in addressing the issue of NCII. Instead, by integrating RLAT, our research aims to shed light on the contextual factors surrounding NCII, offering insights into potential risk factors while focusing on the guilt of those who commit these offenses.

### Research questions and hypothesis

This study seeks to investigate the prevalence and distribution of NCII and its predictors among Italian young adults. Drawing from previous research, we anticipate that individuals with a history of intimate partner violence may be at an elevated risk of experiencing NCII. Additionally, those who engage in sexting may have an increased likelihood of NCII victimization. Moreover, impulsivity and self-disclosure might emerge as risk factors for NCII.

Specifically, the following research questions will be addressed:

1 What is NCII prevalence among Italian young adults, and how does it vary by demographic and social factors?
2 Do individuals who have experienced NCII victimization engage in specific social coping behaviors, such as seeking support from friends or family, or confiding in someone they trust?
3 How do IPV, sexting, self-disclosure, and impulsivity interact in predicting involvement as a victim in NCII?

We hypothesize that individuals with a history of intimate partner violence may be at a higher risk of becoming victims of NCII (*H1*). We further hypothesize an interaction effect between online self-disclosure and impulsivity in decision-making (*H2*).

Individuals with high levels of online self-disclosure and impulsivity may be more prone to impulsive decisions when sharing personal information online, potentially leading to NCII victimization without fully considering the risks and consequences. This study will provide valuable insights into the understanding of NCII in an Italian sample by considering predictive intrapersonal and interpersonal factors that may contribute to the phenomenon.

## Methods

### Participants

We recruited a sample of 2047 Italian adults and young adults by conducting an online survey.

The survey was advertised on various social media platforms, including the project's Facebook page, and it was framed as a study related to NCII (the term "revenge porn" was predominantly utilized in our advertisements, primarily because it is a widely recognized and familiar term among the public).

Additionally, the survey link was shared with influencers and featured in online magazines and interviews, employing a snowball sampling approach. Participants were not compensated for their participation.

Among the respondents, 29.3 % identified as female, 53.4 % as male, and 16.9 % did not specify their gender. The average age of our participants was 19.10 years (SD = 14.4, range 18 - 68). Moreover, 12.3 % of the study participants self-identified as sexual minorities.

Overall, 9.4 % of the respondents completed compulsory education, 33.7 % finished high school, and most (48.4 %) had a higher education title. Almost half of the participants lived in Northern Italy (44 %), while 15.1 % lived in the Center regions and 22.5 % lived in the southern regions. 7.1 % of participants had an immigrant background (i.e., not born in Italy and/or parents not born in Italy).

### Measures

To examine IPV, participants were asked to answer 13 questions based on their experiences on a 5-point Likert scale (1 = Never;2 = Once; 3 = Sometimes; 4 = Almost once per month; 5= More than once per month; Barter et al., 2009; Radford et al., 2011). The original scale was translated into Italian by a professional and then back-translated by a native English speaker to ensure accuracy. In our sample, McDonald's Omega for the scale showed excellent reliability (Ω= .95). Different typologies of intimate violence, such as psychological/emotional violence and cyberstalking, were analyzed to examine the experiences associated with them (e.g., "He/She screamed at me," "He/She used physical strength to hurt me").

Disclosure in social networks was measured using a sub-scale of 10 items from the adaptation of the Self Disclosure Index (Lyvers et al., 2020), asking to respond how often they posted online personal information on a Likert scale from 1 to 5 (1=Never: 2=Rarely; 3=Sometimes; 4=Often; 5=Very often; e.g., "I post about my deepest feelings," "I post about my fears"). For this subscale, the McDonald's Omega obtained was .92. To ensure accuracy, the scale was professionally translated into Italian and back-translated by a native English speaker.

In order to measure impulsivity in our study, the Lack of Premeditation subscale from the Italian UPPS-Short questionnaire was used (Cyders et al., 2014; Italian validation by Donati et al., 2021). The four items were scored on a Likert scale from 1 to 4 (1 = *I* completely disagree: 2 = *I* disagree; 3 = *I* agree; 4 = *I* completely agree;) showing good internal consistency (Ω= .78; e.g.,"I usually carefully reflect before doing something (R)," "Sometimes I enjoy doing frightening activities").

Sexting was measured by asking six questions measured by the Experiences of Sending and Receiving Sexual Images Likert scale, from 1 to 4 (1=Never, 2=Once, 3 = A few times, 4=Often; (Stanley et al., 2018). To ensure accuracy, the scale was professionally translated into Italian and back-translated by a native English speaker. Participants were asked if they had ever texted, called, or used social media to send a sexual message or image to a partner or ex-partner. The sub-scale showed good reliability (Ω= .79).

To measure NCII victimization episodes, we assessed the responses to threats, disclosure, and reporting of NCII incidents, frequency and duration of threats according to an adapted 12 items Likert scale (1=Never; 2=Once; 3=More than once; Ω= 0.86; e.g., "Somebody tricked me into forwarding him the images," "Somebody activated my webcam"; Wolak and Finkelhor, 2016). Given the unavailability of an Italian version of the original scale, a professional translator was employed to translate it into Italian. The translated version was then back-translated by a native English speaker to ensure accuracy.

The assessment of social coping strategies in response to NCII was conducted using an exploratory approach. Rather than using a validated scale, participants were presented with a series of multiple-choice questions to explore the diverse coping mechanisms employed post-victimization. This approach provided a comprehensive view of coping strategies, such as seeking support, confiding in trusted individuals, or connecting with others who had similar experiences.

### Procedure

The online questionnaire was part of the CREEP Project *"Trust me, it's for me. Criminalizing Revenge Porn"*, a publicly funded interdisciplinary research project studying the phenomenon of non-consensual sharing of intimate images and videos, funded by the Free University of Bozen.

This study has been reviewed and received ethical approval from the Ethical Committee of the Free University of Bozen to ensure that it complied with the ethical standards for research (*Code: 2020–11, 4/03/2021*). Participants in the study were required to sign both an Informed Consent and a Privacy Notice before the start of the questionnaire. Only adults (older than 18) were recruited for the study sampling.

The data for the survey was collected using Qualtrics survey software (2022). Data were collected from April 2021 to July 2022.

### Statistical analysis

To better understand the prevalence and characteristics of NCII, we utilized descriptive statistical analysis methods. Specifically, we calculated frequencies and percentages to measure the occurrence of NCII victimization among our study participants. A series of chi-squared tests was employed to examine group differences.

Furthermore, we employed multiple regression analysis to test the hypothesis that specific factors such as IPV, online self-disclosure, impulsivity, and sexting were related to the likelihood of experiencing NCII as victims.

The multiple regression analysis used in the present study allowed us to examine the unique contribution of each independent variable in predicting NCII experiences while controlling for the other variables in the model. In addition to examining the main effects of each variable, we also explored potential interactions between variables in our analysis. Our hypothesis suggested that the combination of multiple risk factors, such as self-disclosure and impulsivity, may have a more significant impact on the likelihood of experiencing NCII than the risk factors considered independently. As such, we tested interaction effects between designated variables to assess the impact of different combinations of independent variables on NCII experiences. All the analyses presented in this study were performed using the R software (v4.2.2, R Core Team, 2022).

## Results

### Descriptive statistics

#### Sexting

Overall, 33.9 % of respondents reported having sent sexually explicit content of themselves once during or after a relationship. The adjusted residual chi-square test suggests a significant association between gender and sexting (*F* = 60.9 %, *M* = 20.1 %, χ2 = 386.06, *df*=1, *p*<.001), showing that females were three times more likely to have engaged in sexting than males. Moreover, sexual minorities reported a higher rate of sexting (56.7%) compared to heterosexuals (29.1%).

About 11.4 % of the sample reported that they had sexted only once, while the rest said they had exchanged sexual content multiple times. Additionally, 8.1 %(*N* = 167) of participants reported exchanging sexual content during lockdown periods (COVID-19) more than usual.

Among those who reported having sexted at least once, almost half of them (41.4 %) reported being forced to sext against their will. This was true both for males (42.7 %) and females (41.8 %).

#### Forms of non-consensual intimate image diffusion

As shown in Table 1, about 6.8 % of participants reported that they had been threatened with NCII ("Has it ever happened that someone has threatened to spread one or more of your images or videos with sexual content?").

**Table 1:** Prevalence of different NCII threats.

		% (count)
Have you ever been threatened of NCII?		
	Yes	6.8 % (141)
	No	92.6 % (1896)
What do you think the aggressor wanted by threatening you?		
	Obtain other explicit pictures	11.3 % (16)
	Force me to stay with him/her or to go back with him/her	18.4 % (26)
	Meet offline to have sex	2.8 % (4)
	Have online sex	2.1 % (3)
	Extort money from me or my family	18.3 % (22)
	Obtain explicit pictures of others (i.e., friends) without their consent	1.4 % (2)
	Hurt me as revenge for something already happened	15.6 % (22)
	Have fun with his/her frinds	8.5 % (12)

A significant percentage, representing 18.4 % of victims, perceived the threat as an effort by their partner or ex-partner to either exert control within the relationship or to rekindle it. Most respondents reported that the threat was a form of monetary extortion (18.3 %).

Moreover, 15.6 % of victims stated that the threat was a form of revenge for something that happened in the past.

In our sample, 3.3 % of participants declared that someone had shared intimate sexual or erotic images without consent. A chi-squared test was run to examine the association between the threat of NCII and effective NCII victimization in our study sample. The results showed a significant association between the two variables (χ2 = 274.67, *df*=1, *p* < .001). This finding suggests that the threat of NCII may be an essential predictor of victimization. Furthermore, a series of chi-square tests of association between the gender (male, female) or sexual orientation (heterosexual, sexual minorities) of the victim and the reported victimization was run. The relationship with gender was not significant (*p* = .164). Nonetheless, an association between the victimization and the sexual orientation of the victim was found (*χ*^2^ = 10.16, *df*=1, *p* < .001), showing that individuals who identify themselves as belonging to a sexual minority were more likely to be victims of NCII than their heterosexual counterparts.

Regarding the type of content, the most common NCII victimization concerned genitals or nudity (52 %), while 24 % of them depicted a sexual act (intercourse or masturbation).

Table 2 shows the prevalence and incidence of different experiences of NCII in participants who self-reported victimization (*n* = 66). The most prevailing experience of NCII was through initial voluntary content sharing, with 46.2 % of the victims spontaneously providing the images to their partner during a relationship. Moreover, sextortion and psychological pressure were experienced by 25.3 % of the victims. In particular, 19.4 % of the victims were involved in severe sextortion ("Someone forced me to send him/her pictures").

**Table 2:** Prevalence of different types of NCII among victims.

		Total % (count)	Males % (count)	Females % (count)	Heterosexuals % (count)	Sexual Minorities % (count)
**Did someone share sexual/intimate pictures of yours without your consent?**						
	Yes	3.3 % (66)	2.8 % (38)	4.3 % (28)	2.7% (50)	6.9% (16)
	No	96.7% (1911)	65.7 % (1300)	30.8 % (611)	97.2 % (1752)	93.1 % (213)

**Please indicate how often someone has come in possession of sexual images of you and then shared them in the following ways.**						
					**Once**	**More than once**

I spontaneously provided the images for an erotic game between us within our relationship, and I passed them on to him/her.					26.8 %	46.2 %
Someone coerced me to send him the images/videos, and it made me feel bad or guilty if I didn't send them to him/her.					25.3 %	25.3 %
Someone tricked me into sending him/her the pictures.					32.8 %	19.4 %
Someone forced me to send him/her pictures.					19.4 %	19.4 %
Someone told me he/she would pay me for my images.					17.9 %	
I thought the images were for a job reason (e.g., model, casting).					16.4 %	5.9 %
Someone recorded with the webcam without me noticing or without my consent.					28.3 %	17.9 %
Someone activated my webcam from remote.					14.9 %	8.9 %
Whoever disclosed the image received it from other people who shared it voluntarily.					20.8 %	22.3 %
The person who disclosed the image received it by hacking the devices of other non-consenting people (e.g. opening another person's mobile phone and stealing the images).					17.9 %	10.4 %
Someone created fake or photoshopped images.					28.3 %	8.9 %
Someone hacked into my devices (laptops, mobile phones, computers, etc.) or in my online accounts to steal my images.					23.7 %	13.1 %

Overall, 22.6 % of the victims reported having experienced three types of NCII, 10.4 % four types, and 6.6 % five or more types of victimization.

Almost three-quarters of victims (72.7 %) of our sample knew the aggressor. In most cases, victims had known the aggressor for over a year (42.4 %), while 18.1 % had from three months to a year. In 13.6 % of the cases, the aggressor was a friend or someone close to the victim, while in 7.5 % of cases, it was a co-worker (Table 3). Moreover, in 51.1% of the cases, the perpetrator was identified as a former partner.

**Table 3:** NCII descriptive statistics: relationship of the victim with the aggressor.

		Total % (count)	Males % (count)	Females % (count)	Heterosexuals % (count)	Sexual Minorities % (count)
Did you know the aggressor?						
	No	27.2 % (18)	13.1 % (15)	10.7 % (3)	32 % (16)	12.5 % (2)
	Yes	72.7 % (48)	60.5 % (23)	89.2 % (25)	68 % (34)	87.5 % (14)
How well did you know the aggressor?						
	I knew him/her for a little time (less then 3 months)	18.1 % (12)	15.7 % (6)	21.4 % (6)	18 % (9)	18.7 % (3)
	I knew him/her quite well (more than 3 months)	18.1 % (12)	21 % (8)	16.114.2 % (4)	14 % (7)	31.2 % (5)
	I knew him really well (more than a year)	42.4 % (28)	23.6 % (9)	53.5 % (15)	36 % (18)	37.5 % (6)
Wich kind of relationship did you have with the aggressor?						
	Partner (current/former)	45.4 % (30)	31.5 % (12)	64.2 % (18)	46 % (23)	81.2 % (13)
	Current partner	10.6 % (7)	15.7 % (6)	(0)	8 % (4)	12.5 % (2)
	Former partner	51.1% (34)	31.5 % (12)	64.2 % (18)	38 % (19)	68.7 % (11)
	Stable partner	28.7 % (19)	26.3 % (10)	28.5 % (8)	26 % (13)	31.2 % (5)
	Casual partner	31.8 % (21)	21 % (8)	35.7 % (10)	20 % (10)	50 % (8)
	Relative	1.5 % (1)	2.6 % (1)	0	0	6.2 % (1)
	Friend	13.6 % (9)	7.8 % (3)	10.7 % (3)	0	37.5 % (6)
	Co-worker	7.5 % (5)	2.6 % (1)	14.2 % (4)	2 % (1)	25 % (4)
	Someone I only met online	(0)	(0)	(0)	(0)	(0)
	Someone I met both online and offline	1.5% (1)	(0)	(0)	(0)	(0)
Total		66	38	28	50	16

#### Coping with NCII

Participants in our sample who reported being a victim of NCII also completed a further session in the questionnaire regarding coping strategies adopted to face these negative experiences. Among those who reported NCII experiences, 56.7 % spoke with somebody about what happened. The majority of them choose a close friend to share the negative experience. While only one participant reported having contacted anti-violence support centers, none of the victims contacted the authorities (police), and only one contacted Social Media Help Centers. Moreover, the majority of sexual minority victims preferred not to speak with anyone (62.5%), in contrast to heterosexuals (38%) who were less inclined to do so.

As reported in Table 4, 43.9 % of the victims did not talk with anyone about the negative experience. Among them, 37.9 % chose not to talk about what happened because they were too embarrassed. Most victims who preferred not to speak with anyone favored an avoiding coping strategy since 13.7 % thought talking would not have helped, and 6.8 % would act as if nothing had happened.

**Table 4:** Prevalence of different social coping strategies among victims of NCII.

		% (count)
Have you spoken with someone?		
	Yes	56.7 % (37)
	No	43.9 % (29)
With whom?		
	A friend	34.2 % (13)
	My parents	2.6 % (1)
	Relatives	2.6 % (1)
	Non-family member	2.6 % (1)
	Anti-violence support center	2.6 % (1)
	Police	(0)
	Priest	(0)
	Social media administrator	2.6%% (1)
	Other	5.2 % (2)
Why haven't you spoken with someone?		
	I was too embarrassed	37.9 % (11)
	I was worried I could have got in trouble	(0)
	I thought I could have handled that by myself	3.4 % (1)
	I thought talking with someone would have not helped	13.7 % (4)
	I was worried the aggressor could find that out	6.8 % (2)
	I wanted to act like nothing happened	6.8 % (2)
	Threats stopped before I could ask for help	(0)

### Regression model

To achieve a more profound understanding of the risk factors associated with NCII victimization, we conducted a multiple regression analysis, considering IPV, self-disclosure, impulsivity, and sexting as predictors of NCII, including the interaction effect of self-disclosure and impulsivity, based on our hypotheses. Table 5 presents the results of the multiple regression model.

**Table 5:** Regression analysis of NCII (*N* = 2047).

Predictor	*β*	*β* 95 % CI [LL, UL]	*^SE β^*	Fit
Age	−0.00	[−0.12, 0.12]	0.05	
Gender	−1.81	[−4.16, 0.52]	1.16	
IPV	1.85**	[1.21, 2.50]	0.32	
Self-disclosure	0.57	[−0.41, 1.90]	0.58	
Impulsivity	0.54	[−1.00, 0.86]	0.46	
Sexting	0.98*	[0.14, 1.82]	0.41	
Self-disclosure*Impulsivity	1.05**	[0.23, 1.86]	0.40	
				*R^2^* = 0.638**
				95 % CI [.47,.74]

*T Note.* A significant *β* -weight indicates the beta-weight and semi-partial correlation are also significant. *β* indicates the standardized regression weights. *LL* and *UL* indicate the lower and upper limits of a confidence interval, respectively. * Indicates *p* < .05. ** indicates *p* < .01.

The fitted regression model predicted 63.8 % of the variance with a significant effect (*R*^2^=0.638, *F* = 16.3, *p* < .001). We used the Variance Inflation Factor (VIF) test to examine multicollinearity among the predictor variables. The mean VIF was 1.9, with a range of 1.3 to 2.7. As a cutoff value of 5 is commonly used to indicate the presence of multicollinearity, our results suggest that multicollinearity was not a significant issue in this analysis. According to our analysis, both IPV (*β=1.85, p <* .001) and sexting (*β=0.98, p =* .022*)* significantly predicted NCII experiences in victims.

**Fig. 1:**
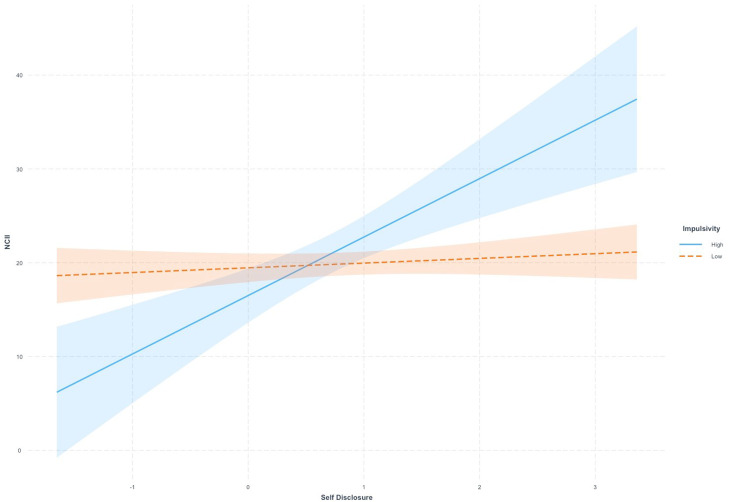
Interaction Effect of Impulsivity and Self-Disclosure Predicting Nonconsensual Intimate Image Diffusion (NCII) Victimization.

While self-disclosure and impulsivity alone had no significant predictive value on NCII victimization, their interaction effect was statistically significant (*β* = 1.05, *p* = .013). To further elucidate this interaction, we dichotomized impulsivity scores based on the median value (11) into low and high groups. Fig. 1 displays the interaction effect of impulsivity on self-disclosure predicting NCII victimization. Notably, self-disclosure was also higher at higher levels of impulsivity, which heightened the risk of encountering NCII victimization.

## Discussion

The present study results from the first extensive investigation of NCII on the Italian population and on behaviors that appear to be strictly related to this phenomenon: sexting, sextortion, and IPV.

Our findings show that many respondents reported engaging in sexting behavior. Moreover, the COVID-19 pandemic appears to have led to an increase in the exchange of sexual content, which may have further exacerbated the risk of NCII victimization. However, many respondents reported pressure from the partner to exchange sexual images, thus highlighting a "gray zone" where personal choice seems to be hindered by abusive dynamics in the couple.

Our results also shed light on the types of threats suffered by the victims. Many victims reported that threats were used as coercion and control within a relationship or for financial gain. Almost one-fifth of victims reported being threatened or forced to send pictures, indicating perpetrators' use of coercion and power to manipulate and exploit their victims.

Concerning NCII, our study shed light on the different forms and prevalence of victimization experienced by our sample population. Although the percentage of victims of NCII seems relatively quite low (3.3 %) compared to other studies (Gámez-Guadix et al., 2022; Patchin & Hinduja, 2020; Valkenburg & Peter, 2011; Walker & Sleath, 2017), it should be considered that victims of NCII reported multiple types of victimization, with over one-third of victims experiencing three or more types of NCII victimization.

In a recent systematic review, the authors struggled to identify common and standard rates of NCII across countries (Walker & Sleath, 2017). It was observed that when trying to summarize the research on the frequency of victimization and perpetration of NCII, it can be challenging to make comparisons due to the absence of a standardized definition of NCII. This leads to challenges in obtaining accurate and comparable data on NCII.

Victims in our sample declared that NCII often started as voluntary content sharing, with a high proportion of them (44.7 %) spontaneously providing images to their partners during a relationship.

Furthermore, our results suggest that a significant proportion of victims experienced NCII victimization combined with sextortion. As our results pointed out, 72.7 % of NCII victims knew the aggressor, with 42.4 % being close to them for more than a year. These results highlight a continuity between the offline and online domains in the risk pathways leading to NCII victimization. Most of the time, the aggressor was not a stranger met online but a well-known person from a trusting relationship/ex-relationship. Half of the time, our respondents identified the aggressor as a partner (current or former). This trend, confirmed by previous research, might result from dysfunctional intra-partner dynamics, such as control and manipulation, lack of boundaries, jealousy, revenge, and unauthorized access to personal contents (Powell et al., 2020; Priebe & Svedin, 2012; Stanley et al., 2018). Despite the common perception that NCII is solely an online phenomenon, our study shows that the experience of offline and online violence intersects and actively affects individuals' lives, highlighting the importance of understanding how exposure to multiple forms of violence may exacerbate the damage of the victim.

The coping strategies adopted by participants who experienced NCII victimization were also examined in our study. We found that many victims chose not to talk to anyone about their experience, citing embarrassment as a significant reason for their reluctance to discuss what had happened. However, close friends were the most commonly chosen confidants among those who did speak to someone. The results obtained from our study indicate that informal support networks may play an essential role in helping victims cope with the emotional and psychological distress resulting from NCII victimization. Nonetheless, few victims sought professional support, indicating the need for increased awareness and availability of support services for NCII victims.

Moreover, our research found a significant difference in the occurrence of NCII between sexual minorities and heterosexuals, with the first at a higher risk of experiencing it (McGlynn et al., 2017; Said & McNealey, 2022; Priebe & Svedin, 2012; Stanley et al., 2018). Sexual minorities appeared as particularly vulnerable group in our sample, pointing out that societal stigma and shame for sexual orientation or gender identity for LGBTQ+ individuals make it harder to report NCII, as they may fear discrimination or not being taken seriously by authorities. This highlights the importance of creating inclusive and safe environments for LGBTQ+ individuals and providing legal protections and resources for those who have experienced NCII.

The current study's findings provide insight into the interplay of the predictors of NCII. Specifically, the multiple regression analysis indicated that sexting and intra-partner violence were significant predictors of NCII. This suggests that the risk of NCII victimization is combined with other forms of abuse, where sexting also can be coerced by the aggressor (Englander & McCoy, 2017; Langhinrichsen-Rohling et al., 2012; Van Ouytsel et al., 2021).

These outcomes align with prior research, which pointed out that sexting was associated with an increased risk of NCII, particularly when combined with other risk factors, such as intimate partner violence (Eaton et al., 2020; Rodríguez-Castro et al., 2021; Walker & Sleath, 2017). One of the reasons why IPV might be a risk factor for NCII is the control and manipulation aspects of IPV, where one partner exerts control over the other; IPV involves a power imbalance, where the abuser holds more power than the victim, and this power imbalance can be used to control and manipulate the victim, including sharing intimate images without consent. This dynamic may also provide the aggressor with easy access to private content. It may facilitate privacy violations, where the abuser may monitor, restrict, or use the victim's contacts to humiliate and damage the victim's reputation as an act of vengeance if the relationship ends against their will.

The analysis performed in this study also showed that the interaction effect between self-disclosure and impulsivity was a significant predictor of NCII. This result is consistent with earlier studies, including the Pratt and colleagues (2014) meta-analysis, which found that low self-control is associated with an increased risk of victimization.

The significant interaction effect between impulsivity and self-disclosure suggests that the relationship between these variables and NCII victimization may be more complex than a linear path. We argue that higher levels of self-disclosure and impulsivity reported among victims may result from a dysfunctional decision-making process induced by stressful experiences like IPV and NCII than an individual propensity for risk-taking behaviors. Moreover, IPV might result in very stressful experiences among the victims and can provoke post-traumatic stress disorder, severely affecting mental health (Dokkedahl et al., 2022). In such a particular stressful context, stress-related experiences may affect the decision-making processes of the victims. A review of decision-making processes under stressful circumstances (Morgado et al., 2015) points out that individuals may have difficulties using emotion regulation strategies under stress, which could lead to deficits in decision-making such as reduced self-control, altered valuation/feedback processing and increased impulsivity in decision implementation: acute stress seems to enhance decision biases, mainly increasing risky choices, following personal characteristics such as gender and ind. We believe this line of investigation might open new perspectives of approach to different neuropsychiatric conditions in which these impairments are central, like in IPV-NCII experiences. Although the research on emotion and decision-making is not in its early stages, to our knowledge, there are really few studies investigating how IPV and digital violence may affect emotional processes and decision-making, thus exposing the victims to further risks after the first episodes of violence (Averdijk et al., 2016; Lerner et al., 2015). Additionally, identifying decision impairments in patients suffering from these stress-related psychiatric disorders could also help devise interventions to reduce stress and develop more adaptive coping strategies as putative interventions to ameliorate behavioral alterations associated with that psychiatric condition.

## Limitations and implications for future research

One limitation of this study pertains to the composition of our sample, which consisted of a convenience sample that may not be fully representative of the Italian population in terms of age and actual experiences with NCII. Research has shown that NCII is particularly prevalent among adolescents (Gámez-Guadix et al., 2022; Patchin & Hinduja, 2020; Valkenburg & Peter, 2011; Walker & Sleath, 2017), a group that is underrepresented in our sample. Therefore, future research is needed to investigate the relationship between NCII, IPV, and their predictors in this population to gain a deeper understanding of NCII in this age group. Additionally, conducting cross-cultural studies might help generalize the effects of the predictors considered in our study, namely sexting, self-disclosure, and impulsivity.

Another limitation of this study is the potential difficulties in self-reporting traumatic experiences, such as NCII and IPV. Individuals who have experienced NCII and IPV may hesitate to disclose their experiences due to fear of retaliation, shame, stigma, or other factors. This can result in underreporting these experiences and limit our findings' accuracy. Therefore, it is relevant to consider self-reported data's limitations and continue developing methods to improve data collection accuracy in future studies.

While we recognize the importance of assessing trauma, stress, and psychological functioning about the observed interactions, these aspects were not directly examined in this study due to limitations in scope and space. Future research should focus on incorporating assessments of trauma, stress, and psychological functioning to gain deeper insights into the mechanisms driving these complex interactions and, ultimately, contribute to more effective interventions that address the root causes rather than solely focusing on survivor behaviors.

Furthermore, our study's data are cross-sectional, and while we applied a statistical model to analyze the data, we cannot establish strictly causal relationships among the variables considered. However, we believe that these preliminary results, which shed light on decision-making processes under stressful circumstances, may reveal potential risk factors for further exposure to violence among victims.

Despite these limitations, our study's methodological approach is a point of strength. We measured actual behaviors as dependent variables rather than relying solely on behavioral intentions or non-ecological experimental designs. This approach allowed for a more accurate representation of the real-life experiences of victims and provided a comprehensive understanding of the phenomenon under investigation. To further enhance the validity and reliability of our results, we recommend that future research consider implementing longitudinal data collection methods to assess changes in behavior over time. This would provide a more detailed understanding of the dynamic nature of NCII victimization and its interplay with IPV, sexting, self-disclosure, and impulsivity.

## Conclusions

In conclusion, this research contributes to understanding the complex and multifaceted issue of NCII and the importance of addressing it from multiple angles. The research presented in this study provides a first understanding of the NCII phenomenon in Italy. Despite its limitations, to our knowledge, this is the first study on a large sample of young adults to be carried out in Italy.

Our research aimed to explore the relationship between NCII, intimate partner violence, and their predictors. While the social and mediatic perception of NCII depicted an image of the event constrained to the digital world, our study confirms that the online and offline experiences of violence intersect to affect people's lives actively. The present findings also highlight the need for future research to further explore the complex interplay between self-disclosure and impulsivity in the context of NCII victimization. The relations between self-disclosure, impulsivity, IPV, and NCII suggest that these factors may cluster together, affecting victims' coping strategies and exposing them to further violence.

In this perspective, counseling after victimization should also consider the effects of violence on decision-making under stressful circumstances induced by multiple forms of violence (physical or psychological, offline and online).

## Research support

This study was funded by the Free University of Bozen, as part of the CREEP project "CREEP Project "Trust me, it's for me. Criminalizing Revenge Porn" (code: BW2080).

## Relationships

The authors declare that they have no known competing financial interests or personal relationships that could have appeared to influence the work reported in this paper.

## Patents and intellectual property

There are no patents to disclose.

## Other activities

There are no additional activities to disclose.

## CRediT authorship contribution statement

**Antonella Brighi:** Conceptualization, Formal analysis, Investigation, Methodology, Project administration, Resources, Software, Supervision, Validation, Visualization, Writing – original draft, Writing – review & editing. **Alberto Amadori:** Data curation, Formal analysis, Writing – original draft, Writing – review & editing. **Kolis Summerer:** Conceptualization, Funding acquisition, Project administration, Resources, Validation, Visualization, Writing – review & editing. **Damiano Menin:** Data curation, Formal analysis, Investigation, Methodology, Supervision, Writing – original draft, Writing – review & editing.

## Declaration of Competing Interest

None.
